# The Life-Cycle of Operons

**DOI:** 10.1371/journal.pgen.0020096

**Published:** 2006-06-23

**Authors:** Morgan N Price, Adam P Arkin, Eric J Alm

**Affiliations:** 1 Lawrence Berkeley National Laboratory, Berkeley, California, United States of America; 2 Virtual Institute for Microbial Stress and Survival, University of California San Francisco, San Francisco, California, United States of America; 3 Department of Bioengineering, University of California Berkeley, Berkeley, California, United States of America; 4 Howard Hughes Medical Institute, University of California Berkeley, Berkeley, California, United States of America; Ivan Matic, INSERM U571, France

## Abstract

Operons are a major feature of all prokaryotic genomes, but how and why operon structures vary is not well understood. To elucidate the life-cycle of operons, we compared gene order between Escherichia coli K12 and its relatives and identified the recently formed and destroyed operons in E. coli. This allowed us to determine how operons form, how they become closely spaced, and how they die. Our findings suggest that operon evolution may be driven by selection on gene expression patterns. First, both operon creation and operon destruction lead to large changes in gene expression patterns. For example, the removal of *lysA* and *ruvA* from ancestral operons that contained essential genes allowed their expression to respond to lysine levels and DNA damage, respectively. Second, some operons have undergone accelerated evolution, with multiple new genes being added during a brief period. Third, although genes within operons are usually closely spaced because of a neutral bias toward deletion and because of selection against large overlaps, genes in highly expressed operons tend to be widely spaced because of regulatory fine-tuning by intervening sequences. Although operon evolution may be adaptive, it need not be optimal: new operons often comprise functionally unrelated genes that were already in proximity before the operon formed.

## Synopsis

In bacteria, adjacent genes are often transcribed together in operons. Which genes are placed together in operons varies greatly across bacteria. This diversity of operon structure can be used to predict the function of genes: genes that are sometimes in an operon are likely to have related functions, even if they are transcribed separately in the organism of interest. However, it has not been clear why this diversity exists or what its consequences are. This work reconstructs evolutionarily recent changes to operon structures in the well-studied bacterium Escherichia coli. Changes in operon structure are shown to be associated with changes in gene expression patterns, so the diversity in operon structure may reflect adaptation to differing lifestyles. Indeed, some of these changes appear to be beneficial to the organism. This work also reconstructs the molecular mechanisms of operon evolution. Understanding these mechanisms should aid other analyses of bacterial genomes. For example, new operons often arise by deleting the DNA between functionally unrelated genes that happen to be near each other. Thus, recently evolved operons should not be used to infer their genes' function. Overall, this work provides a framework for understanding the evolutionary life-cycle of operons.

## Introduction

Operons are groups of genes that are transcribed in a single mRNA. Operons are widespread in all bacterial and archaeal genomes [1–3], and in the typical genome, about half of all protein-coding genes are in multigene operons. Operons often, but not always, code for genes in the same functional pathway [4,5]. Operons are often conserved across species by vertical inheritance [1,2,6,7] and tend to be quite compact: in most bacteria, genes in the same operon are usually separated by fewer than 20 base pairs of DNA [8]. Both conservation and close spacing allow for the computational prediction of operons in diverse prokaryotes [1–3,8,9].

Why are operons so prevalent? The traditional explanation is that genes are placed in the same operon so that they will have similar expression patterns [10]. This also explains why operons tend to contain functionally related genes and why genome rearrangements that would destroy operons are strongly selected against [11]. However, although genes in the same operon do have (mostly) similar expression patterns [12], genes can also be co-regulated without being in the same operon. Thus, it has been argued that co-regulation could more easily evolve by modifying two independent promoters rather than by placing two genes in proximity [13]. In contrast, we argue that for complex regulation, an operon with one complex promoter would arise more rapidly than would two independent complex promoters [14]. As predicted by this theory, operons tend to have more complex conserved regulatory sequences than individually transcribed genes [14,15]. This theory may also be able to explain why some operons, and especially many new operons, contain genes with no apparent functional relationship [4,5,14]—the genes may be required in the same environmental conditions despite being involved in different pathways. For example, some conserved operons contain genes for ribosomal proteins and enzymes of central metabolism, perhaps because both are required in proportion to growth rates [5].

Another popular view has been that operons are selfish: they form because they facilitate the horizontal transfer of metabolic or other capabilities that can be provided by a single operon containing several genes [13]. This theory is consistent with the compactness of operons and also with the observation that operons often undergo horizontal gene transfer (HGT) [13,14,16]. However, essential and other non–horizontally transferred (non-HGT) genes are particularly likely to be in operons [14,17], and non-HGT genes are forming new operons at significant rates [14]. Also, the selfish theory cannot explain the many operons that contain functionally unrelated genes. Thus, it appears that HGT may increase the prevalence of some operons, but that HGT is not the major factor in operon formation.

Finally, it has been suggested that placing genes that code for multisubunit protein complexes in the same operon is beneficial because it speeds complex formation and folding [17,18] or because it reduces stochastic differences between protein levels [19]. Although the most highly conserved operons do tend to code for protein complexes [18], most operons do not; and vice versa, only a few percent of protein–protein interactions involve genes encoded by the same operon [20].

Overall, genome-wide studies have supported the traditional view that operons exist because they facilitate co-regulation. However, many questions about operon evolution remain. For example, genes that are in the same operon in one bacterium are often found in different operons in other bacteria [7]. Do changes in operon structure lead to changes in gene expression patterns, or are genes co-transcribed from one promoter in some organisms and co-regulated from distinct promoters in other organisms, without obvious functional consequences? Are these changes neutral, as suggested by the loss of most ancestral operons in some genomes [7], or are they adaptive? Also, what are the molecular mechanisms behind these changes in operon structure? For example, how do operons form, and how are they destroyed? Why are the genes in most operons so closely spaced, while some highly conserved operons contain widely spaced genes?

**Figure 1:**
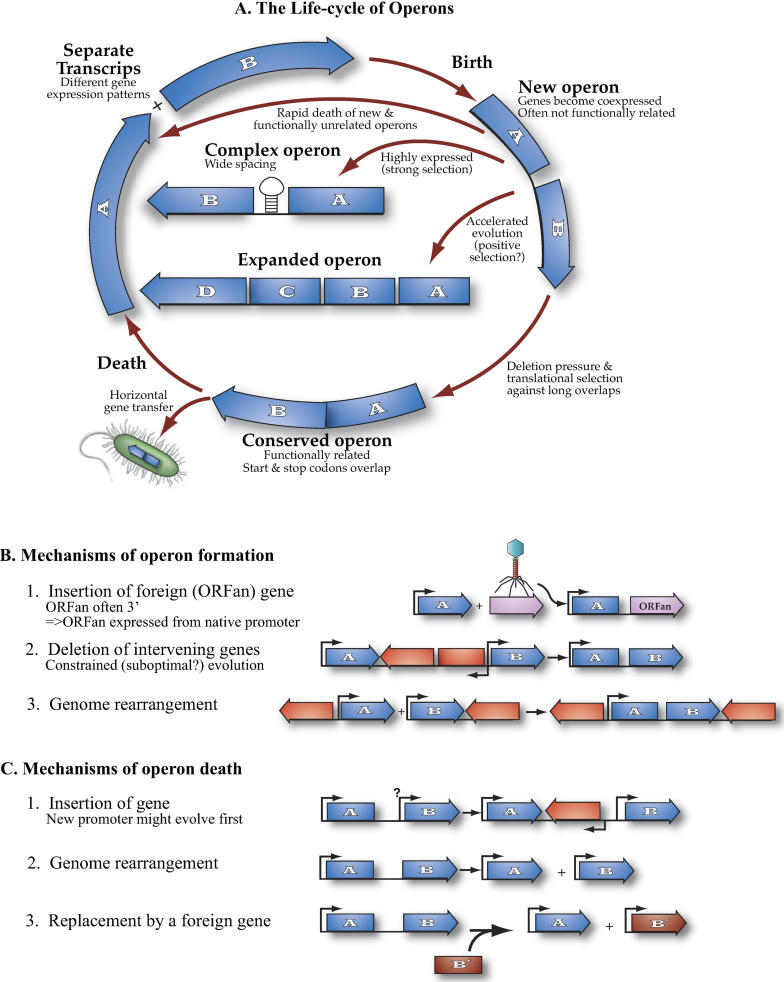
A Model for Operon Evolution

To address these questions, we examined the newly formed or recently deceased operons of Escherichia coli K12. To address the issue of spacing, we compared orthologous operons in E. coli K12 and its close relative Salmonella typhimurium LT2. We also repeated some of our analyses of operon evolution for *Bacillus subtilis.* To summarize our results, we present a model for the life-cycle of operons (Figure 1).

## Results

### How Do Operons Form?

It appears that operons containing native genes form without horizontal transfer events [14], but the mechanism is unknown. Because conserved operons often undergo rearrangements or acquire new genes [7], we distinguish new operons from modifications to pre-existing operons. More precisely, we first examine cases where genes that were not previously co-transcribed are placed next to each other in an operon, and then consider the special case of how new genes are added to pre-existing operons.

#### New operons.

In principle, new operons can form by rearrangement or by deletion. First, genome rearrangements could bring two genes that were not previously near each other into proximity so that they are co-transcribed. Some genomes with large numbers of repetitive elements, such as Helicobacter pylori and *Synechocystis* PCC 6803, have lost most of their ancestral operons, presumably because the repetitive elements cause frequent genome rearrangements [7]. Nevertheless, sequence analysis suggests that H. pylori and *Synechocystis* contain large numbers of operons, and expression data confirms that most of these putative new operons are genuine [3]. Thus, rearrangements may cause the production of new operons as well as the destruction of ancestral operons.

Alternatively, it has been predicted that if two genes are near each other and are on the same strand, then they could form an operon by deleting the intervening DNA [13]. This mechanism is plausible, but as far as we know it has not been tested empirically.

**Figure 2:**
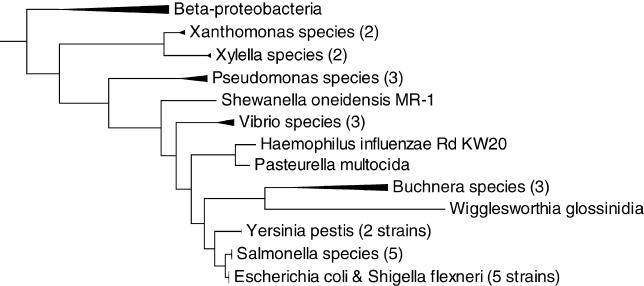
The Relatives of Escherichia coli K12 Considered in This Study Distant relatives (other Proteobacteria, Bacteria, and Archaea) are not shown. The tree is based on highly conserved proteins (see Materials and Methods) and is consistent with that of [42] but contains more taxa.

To identify the mechanism of operon formation, we examined evolutionarily recent operons in E. coli K12. Pairs of adjacent genes were predicted to be in the same operon (or not) from the distance between them on the DNA and the conservation of the putative operon [3]. “Operon pairs” (adjacent genes predicted to be in the same operon) were classified as new if they were conserved only in close relatives [14]. In this study, we considered operons that are new to the Enterobacteria or are shared with somewhat more distant relatives (*Haemophilus, Pasteurella, Vibrio,* or *Shewanella* species—see Figure 2). We also classified genes as native, horizontally transferred (HGT), or “ORFan,” again based on the presence or absence of the gene in other groups of bacteria [21,22]. ORFans are genes that lack identifiable homologs outside of a group of closely related bacteria [23]. Most ORFans are functional protein-coding genes that contribute to the fitness of the organism (they are under purifying selection), and they were probably acquired from bacteriophage [22].

**Figure 3:**
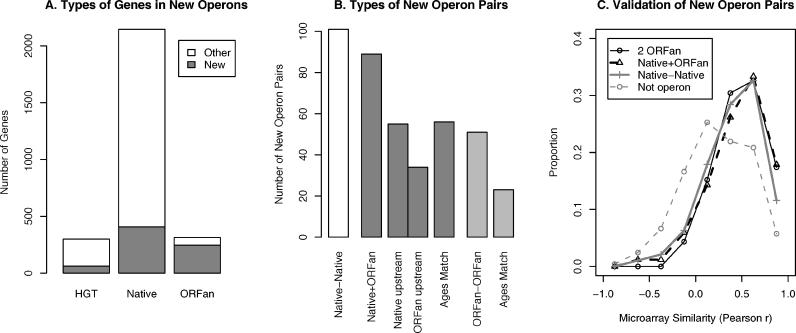
New Operons Often Combine a Native Gene with an “ORFan” Gene That Is Found Only in E. coli and Close Relatives (A) Types of genes in new operon pairs and in other operon pairs. The enrichment for ORFans in new operon pairs is highly significant (*p* < 10^−15^, Fisher exact test). (B) Types of new operon pairs. Only new operon pairs involving native and ORFan genes are shown (there are relatively few HGT genes in the new operons). Within the native–ORFan pairs, we show how often the native gene is upstream of the ORFan, or vice versa. For both the native–ORFan and ORFan–ORFan pairs, we show how often the evolutionary age of the ORFan(s) matches that of the operon. (C) Validation of predicted new operon pairs of each of the three major types. We quantified the similarity of expression patterns in microarray data using the Pearson correlation. As a negative control, we also tested non-operon pairs (adjacent genes on the same strand that are known not to be co-transcribed) from [48].

We found that predicted new operons are highly enriched for ORFan genes (Figure 3A) and often combine an ORFan with a native gene (Figure 3B). A similar pattern was found in B. subtilis (Figure S1). The prevalence of ORFans in new operons is somewhat surprising given that ORFans are less likely than native or HGT genes to be in operons [14]. The most parsimonious evolutionary scenario for constructing a native–ORFan pair is a single insertion event that transfers the ORFan into the genome and places it adjacent to the native gene. To test this hypothesis, we compared the evolutionary age of the new operon to that of the ORFan. The age was determined from the most distant relative that contained the new operon or ORFan (see Materials and Methods). Consistent with the insertion scenario, we found that the estimated evolutionary age of the native–ORFan operon pair often matches the age of the ORFan (Figure 3B and Figure S1B). In *E. coli,* we also found that the ORFan gene is significantly more often downstream of the native gene (Figure 3B; *p* = 0.03, binomial test). This arrangement may be selected for because the ORFan gene is transcribed from a native promoter without perturbing the expression of a native gene. However, in B. subtilis we did not see a significant preference for the native gene to be upstream (Figure S1B).

There are also ORFan–ORFan pairs. For both E. coli and B. subtilis, the age of these pairs often matches the age of both genes in the pair (Figure 3B and Figure S1B), which suggests that the entire operon was imported in a single event. Thus, many of the “new” ORFan–ORFan pairs may actually have been horizontally transferred from a source that has not been sequenced. Because ORFans are suspected to originate in phage [22], that is the most likely source for these ORFan–ORFan pairs. Indeed, because phages have compacted operon-rich genomes, it is surprising that more ORFans are not in such pairs, and that ORFans are less likely to be in operons than other genes [14]. Perhaps the phage operon benefits the phage, whereas only one gene in the operon would benefit the host.

Because new operons are not, by definition, conserved across many genomes, these operon predictions may be less reliable. However, new operon pairs of each of the three major types discussed above tend to have strongly correlated expression patterns (Figure 3C and Figure S1C). Therefore, most of these predictions are probably accurate.

If new operons containing ORFans often form by insertion, how do new operon pairs containing only native genes form? Although we cannot examine the ancestor of E. coli that formed the new operon, we can examine the gene order in close relatives that lack the operon. Specifically, we examined new operon pairs that were shared by E. coli K12, *Salmonella* species, and other Enterobacteria, but had non-adjacent orthologs in *Vibrio* species, which are more distantly related (Figure 2).

Among the ten E. coli operon pairs that have orthologs in *Vibrio* that are not adjacent to each other, we identified six cases where the *Vibrio* genes are near each other (Table 1). Within each of these *Vibrio* pairs, the genes are on the same strand. For four of these pairs, the intervening genes are on the opposite strand, so we are confident that these are not operons in *Vibrio*. (In principle, operons could contain within themselves transcripts on the opposite strand, but this has never been observed in E. coli [9]. In rare cases, B. subtilis operons contain within themselves another transcript on the opposite strand, but this is only possible because B. subtilis has relatively weak rho-dependent termination [24].) For these six new operon pairs, the most parsimonious scenario is that the operon formed by deleting the intervening genes (one event). Because these operons are unique to the Enterobacteria, insertion within the operon in the ancestor of *Vibrio* would require two events (operon formation followed by insertion). Other features of these pairs, such as the absence of homologs for the intervening genes in the Enterobacteria, are consistent with deletion (see Protocol S1). For example, in *E. coli, btuB* and *murI* overlap and are co-transcribed, and the 23 N-terminal amino acids of *murI* are encoded by the 3′ end of *btuB*. These residues are not present in bacteria that lack the operon (unpublished data; evidence that the predicted start codon for *murI* is correct is discussed in Protocol S1). This overlap suggests that the operon formed by deletion in a single event that destroyed the original ribosome binding site of *btuB* as well as other intervening DNA such as promoters and terminators. In general, however, it is possible that the deletion involves several steps (e.g., perhaps the upstream gene's terminator is lost first, and the downstream gene's promoter is lost later).

**Table 1:**
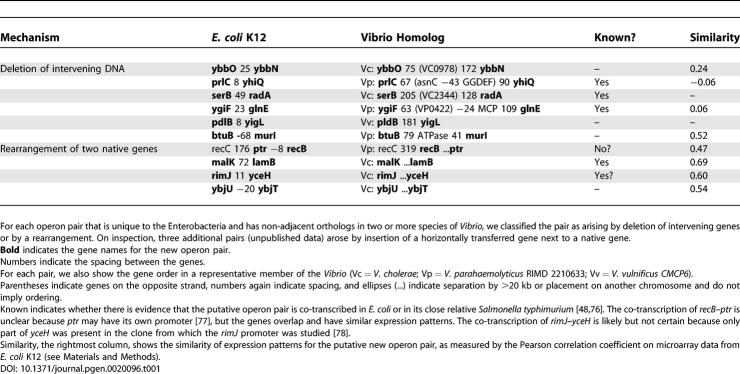
Mechanism of Formation for New Native–Native Operon Pairs

In another four cases, the *Vibrio* genes were distant from each other, so we suspect that the E. coli operons formed by rearrangement (Table 1). In general, we cannot rule out more complicated scenarios that involve deletion, such as: 1) a rearrangement that placed the genes in proximity that was then followed by a deletion; or 2) a rearrangement in the ancestral *Vibrio* that masked the pre-existing proximity of the genes. However, for *ptr–recB,* we can rule out deletion, as the native gene *ptr* was inserted into and destroyed the ancestral operon *recC–recB*.

In summary, operons containing native genes form both by deleting intervening genes and by rearrangements that bring more distant genes into proximity. In contrast, many new ORFan–native operons probably arise from the insertion of the new gene, and often allow expression of the ORFan gene from a native promoter.

#### Modifications to pre-existing operons.

**Figure 4:**
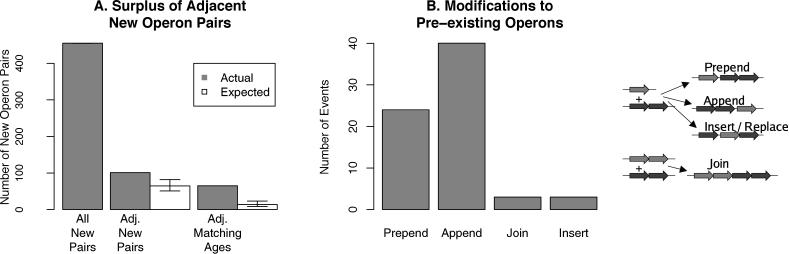
Accelerated Evolution of Some Operons (A) New operon pairs are more likely to be adjacent to each other than expected by chance. The surplus of adjacent pairs of the same age is particularly striking. The error bars show 95% confidence intervals from a c^2^ test of proportions. The model for random evolution is detailed in Materials and Methods. (B) The frequency of different types of modifications to pre-existing operons. The excess of append over prepend pairs is not quite statistically significant (*p* = 0.06, binomial test).

We examined the new operon pairs—adjacent genes that are predicted to be in the same operon in E. coli K12 but transcribed separately in related bacteria—for modifications to pre-existing operons (see Materials and Methods). Such modifications appear to be much less common than the formation of new operons: we identified 455 new operon pairs but only 81 modification events. However, in a surprisingly large number of cases, two or more new operon pairs are adjacent and furthermore of the same age, so that the operon has undergone rapid evolution (Figure 4A). This was also observed in B. subtilis (Figure S2). Adjacent new operon pairs of the same age occur significantly more often than under a completely random model of operon evolution (see Materials and Methods). In other words, some operons are evolving more rapidly than the average operon. Although it is possible for insertions within pre-existing operons to create two or more new operon pairs with a single event, insertions are much less common than additions at the beginning or end of pre-existing operons (Figure 4B). Also, there is a slight preference for appending a new gene to the end of a pre-existing operon instead of prepending a gene to the beginning (Figure 4B), so that the majority of genes retain the original promoter instead of acquiring a new one.

To confirm that some operons are undergoing rapid evolution, we manually examined the modified operons in E. coli. The complete results of this analysis are given in Table S1. We found many cases where two or more changes had occurred to the original operon(s). For example, the older operons *yiaMNO* and *sgbHUE* have joined together with several additional genes to give the known E. coli operon *yiaKLMNO–lyxK–sgbHUE*. Another striking event is the combination of the ancient *sdhCDAB* and *sucABCD* operons, which code for adjacent steps in the TCA cycle, together with an ORFan gene, to give the experimentally characterized E. coli operon *sdhCDAB–b0725–sucABCD*. We also observed several cases where a single gene in an operon has been replaced by a non-homologous gene (Table S1). This supports a previous finding that genes in operons are occasionally replaced by horizontally transferred homologs that are too diverged for homologous recombination to occur [16], although the mechanism by which genes can be replaced or inserted into operons remains unclear.

As the modified operons are evolving more rapidly than the average operon, we considered that these operons might be under positive selection. Proof of positive selection is provided by evolution that is faster than the neutral rate (for example, when changes in a protein-coding sequence that change the protein sequence are more likely than other changes). Unfortunately, the neutral rate of operon evolution is not known, and so we do not see how to perform an analogous test for adaptive operon evolution.

Instead, we tested whether rapid evolution of these operons could be due to weak selection. First, under a neutral model, the ages of the adjacent new operon pairs should be independent, whereas we found that they have a significant tendency to match. Second, we reasoned that if these operons were under weak selection, then the protein sequences of their genes would be evolving rapidly. Instead, we found that genes in modified operons have about the same average level of amino acid identity between E. coli and *Salmonella enterica Typhi* as other genes (88.6% versus 89.3%, *p* > 0.3, *t* test). Adjacent new operon pairs (i.e., runs of three or more genes in new operons) contain genes that are perhaps slightly less conserved on average than other genes (86.1% versus 89.3%, *p* = 0.07), but the effect is small and reflects the modest tendency for genes in new operons to be less conserved that other genes (85.9% versus 89.6%, *p* = < 10^−12^). As the rapid evolution of these operons does not seem consistent with neutral processes, it may result from positive selection.

### Spacings within Operons

#### Close spacings.

Genes in the same operon are usually separated by 20 bases or fewer of DNA [8]. Furthermore, the stop codon of the upstream gene often overlaps the start codon of the downstream gene [25], which gives the impression that the genes are packed together as tightly as possible. Why are genes within operons so closely spaced? Close spacing could arise without selection because of the bias of bacterial genomes toward small deletions [26]. Alternatively, close spacings may be preferred because of translational coupling—the ribosome can move directly from the upstream gene's stop codon to a downstream gene's start codon, which can increase translation from the downstream gene and may also ensure that similar amounts of protein are made from the two genes (reviewed by [27,28]). Translational coupling can apply to any close spacing, and does not necessarily explain why the “canonical” overlaps of 1 and 4 bases, in which the start and stop codons overlap, are so common.

**Figure 5:**
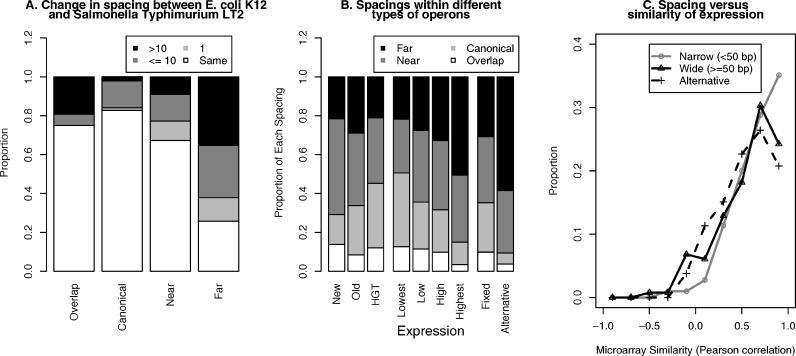
Spacings between Adjacent Genes in the Same Operon (A) Known operon pairs in E. coli often have different spacing than the orthologous operon in Salmonella typhimurium LT2. For each class of spacing in E. coli (*x-*axis), a vertical bar shows the proportion with various amounts of change. (B) The frequency of different types of spacings for operon pairs classified by their evolutionary history (left), their expression level as estimated from microarray data (middle), or whether the operon has an alternative transcript (right). Because operon predictions rely heavily on spacing, only known E. coli operons were used. (C) The distribution of microarray similarity for known operon pairs spaced by less than 50 bp or by more than 50 bp and for alternatively transcribed operon pairs. Operons that are known to be alternatively transcribed were excluded from the “narrow” and “wide” sets.

To study the evolution of close spacing, we first compared spacings within orthologous operons between E. coli K12 and its closest relatives. Because spacing is a major factor in operon predictions, we examined only experimentally characterized operons. The spacing within operons evolves very rapidly—in the close relative S. typhimurium LT2, a large minority of spacings has changed (Figure 5A). Even in another strain of *E. coli, E. coli* O157:H7, where the typical protein is 99.5% identical in sequence to E. coli K12, 6.4% of spacings have changed. The changes in spacing are not artifacts from errors in predicted gene starts: if they were, then the change in spacings would often be a multiple of three, but only 34% of changes in spacings between E. coli and *Salmonella* are by a multiple of three, which is indistinguishable from the 33% that would be expected by chance. Canonical spacings are also often different between E. coli and *Salmonella* or between B. subtilis and its close relative Bacillus licheniformis (Figure 5A and Figure S3A), which suggests that canonical spacing may not be under strong selection.

To see how canonical spacings form, we compared the DNA sequences of operon pairs that are at canonical spacings in E. coli but not in *Salmonella,* or vice versa (Table 2). The canonical overlap of the start and stop codons can easily form by deletion (Table 2). Spacing changes are often accompanied by small insertions or deletions at the ends of the protein sequences (e.g., *cysNC*); we speculate that these protein sequence changes are neutral. We also noticed that canonical overlaps can easily turn into larger overlaps by disrupting the stop codon *(cysNC* and *cstC*–*astA)*. These results are consistent with previous reports that overlapping genes often form by disrupting the upstream gene's stop codon and that this sometimes results in the addition of new coding sequence [29,30]. Because greater overlaps are less common than the canonical overlaps, at least for old operons (Figure 5B), this also suggests that there is selection against greater overlaps. Greater overlaps can eliminate translational coupling (reviewed by [28]) or they might otherwise interfere with translation. New operons are significantly less likely to be at the canonical spacings than are old operons (Figure 5B; *p* < 0.01, Fisher exact test), which is consistent with the idea that canonical spacings form by deletion after the operon has already formed.

**Table 2:**
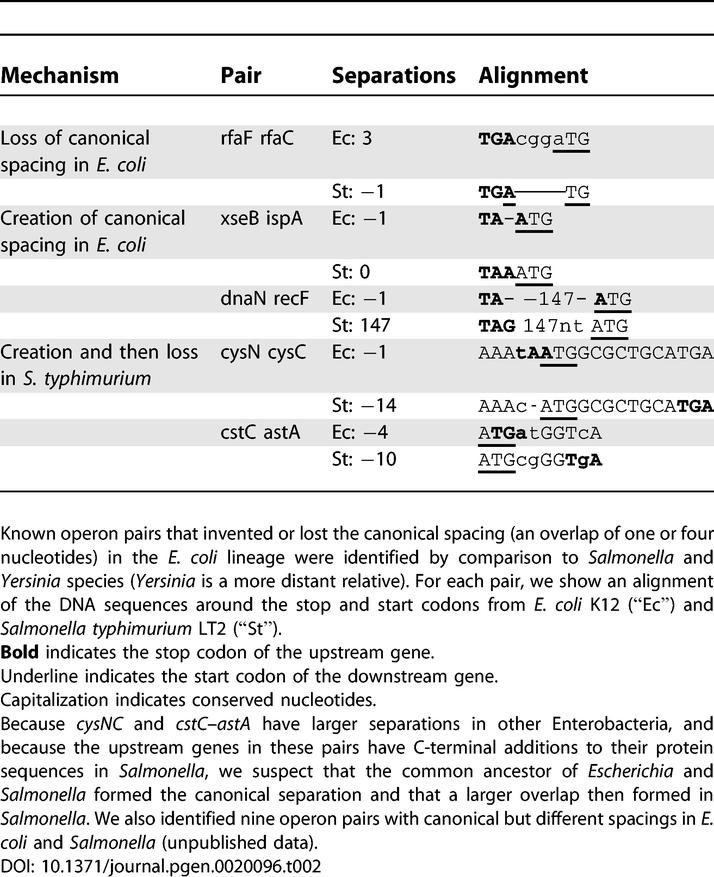
Mechanisms for Forming the Canonical Spacing

It has also been suggested that the canonical spacing might be common because it stabilizes the transcript—with such close spacings, there is no intergenic region that is free of ribosomes and exposed to RNAses [8]. To test this hypothesis, we examined three genome-wide datasets of mRNA half-lives [31,32]. Operon pairs with canonical separations tended to have slightly longer half-lives for both downstream and upstream genes in all three datasets, but the effect was not consistently statistically significant (unpublished data). We concluded that spacing is not a major determinant of mRNA half-lives and that transcript stability is unlikely to explain the prevalence of overlapping start and stop codons.

Overall, we argue that canonical overlaps form by neutral deletion and are maintained by selection against greater overlaps. However, changes to the spacing are likely accompanied by changes to the translation initiation rates of the downstream gene (e.g., switching to a new Shine–Dalgarno sequence or modifying translational coupling). We would expect these changes to expression levels to be under selection. Indeed, in laboratory experiments, the expression level of the *lac* operon evolves to optimality in a few hundred generations [33]. Thus, changes in operon spacing could reflect fine-tuning of expression levels.

#### Wide spacings.

Although genes in operons tend to be closely spaced, genes in highly expressed operons, as identified by codon adaptation, tend to be widely spaced [25,34]. We confirmed with microarray data that highly expressed operons in E. coli and B. subtilis often have wide spacings of more than 20 base pairs (see middle of Figure 5B and Figure S3B). The correlation of spacings with mRNA levels is stronger than with codon adaptation (unpublished data)—we suspect that this is because the empirical mRNA levels are less noisy estimates of expression levels than codon adaptation (see Materials and Methods). The wide spacings within highly expressed operons seem surprising, both because they reduce translational coupling [28] and because the additional RNA in highly expressed transcripts would waste the cell's resources. However, wide spacings are particularly common for alternatively transcribed operon pairs that have internal promoters or terminators (Figure 5B and Figure S3B).

To see if the sequences between the widely spaced E. coli operon pairs contain functional sequences, we examined phylogenetic footprints (conserved putative regulatory sequences) from McCue et al. [35]. 29% of the intergenic regions between known operon pairs that are separated by 50 or more bases contained phylogenetic footprints, which is statistically indistinguishable from the proportion of 38% for known alternative transcripts (*p* > 0.5, Fisher exact test). These conserved sequences averaged a total of 37 bases per pair (median 32), which is considerably larger than Shine–Dalgarno sequences. We searched the literature for evidence of function for the first 15 pairs with footprints, and found five attenuators or partial terminators, three internal promoters, two translation leader sequences, one small RNA not included in our database, two conserved REP sequences of unknown function, and only two cases with no information in the literature. Thus, most of these footprints correspond to functional regulatory sequences, and by extension, most widely spaced operons are subject to complex regulation. Consistent with this claim, widely spaced operons have significantly less similar expression patterns than do narrowly spaced operons, even if they are not known to be alternatively transcribed (Figure 5C; *p* = 0.002, *t* test). Instead, their similarity of expression is about the same as for pairs that are known to be alternatively transcribed (Figure 5C; *p* > 0.5, *t* test). This suggests that unidentified alternative transcripts are very common in E. coli. In *B. subtilis,* most experimentally identified operon pairs already have known alternative transcripts if they are widely spaced (unpublished data). Thus, in both organisms, wide spacings indicate complex regulation. The correlation of these wide spacings with expression levels suggests regulatory fine-tuning, because making unnecessary proteins would be more costly in materials or energy or more deleterious in undesired protein activity if the proteins are highly expressed.

### Death of Conserved Operons

Because few operons are conserved across all or even most bacteria [7], it is clear that after operons form, many of them “die.” Operons could be lost by the deletion of one or both genes or else by splitting the operon apart. Here, we focus on cases where a conserved operon has split apart, so that E. coli retains both genes but they are not in the same operon. In particular, we ask by what mechanisms the operons die, and whether certain types of operons are more likely to die.

#### Operon death by insertion, rearrangement, and replacement.

To identify dead operons in E. coli K12, we first analyzed the predicted operons in its relatives. We considered conserved operon pairs that were predicted in more than one group of related bacteria and for which orthologous genes were present in E. coli. To avoid cases of unclear orthology, we required both genes to be the only members of their respective COGs (conserved orthologous groups [36] in E. coli K12 (see Materials and Methods). We then asked whether these E. coli K12 genes were in the same operon. Using these criteria, we identified 66 dead operon pairs that were split apart and 334 live operon pairs that were still co-transcribed.

When we examined the functions of these dead operon pairs, we found 15 functionally related dead operons and six functionally unrelated genes that are probably growth-rate regulated (Table 3). Growth-related genes are often found together in operons even when there is no close functional relationship [5]. Of the remaining dead operon pairs, 16 are functionally unrelated and 29 contain uncharacterized genes.

**Table 3:**
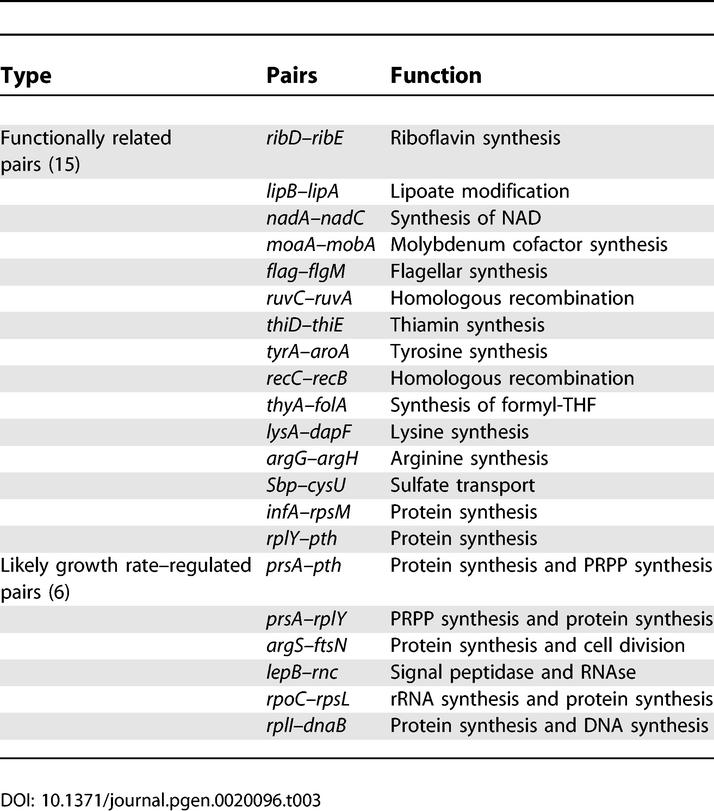
Dead Operon Pairs Comprising Functionally Related Genes or Likely Growth-Regulated Genes

For 11 of the 66 dead operon pairs, the genes are still near each other on the chromosome. In these cases, the operon was probably destroyed by an insertion event. For example, the insertion of *ptr* discussed in a previous section appears to have both created the new *ptr–recB* operon pair and destroyed the ancestral *recCBD* operon. In the other 55 cases, the operon may have been destroyed by genome rearrangements. For example, the dead operon pair *yebI–yebL* is divergently transcribed in E. coli, which strongly suggests that the operon was destroyed by a local inversion.

**Figure 6:**
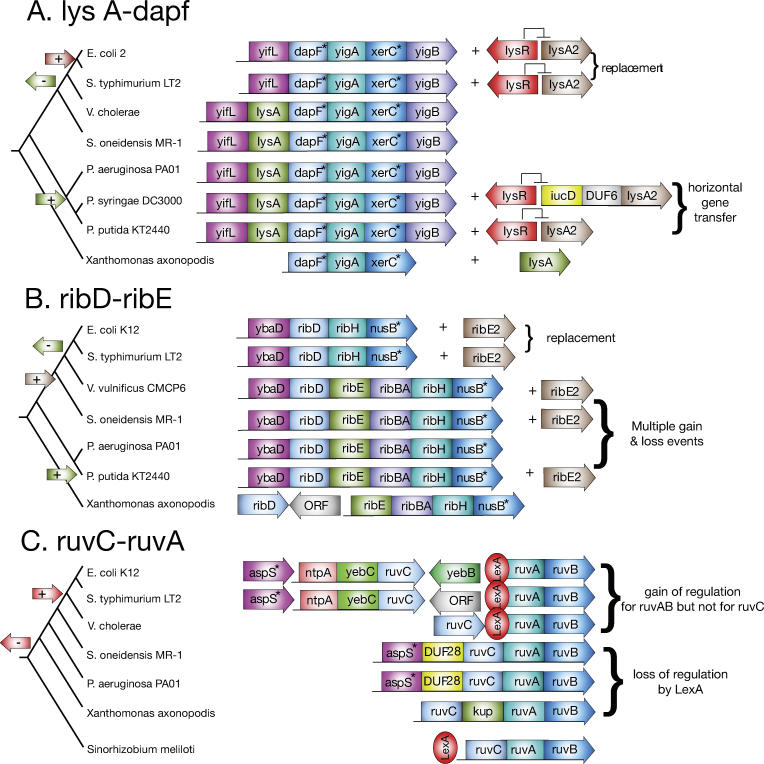
Reconstructed Histories of Three Dead Operons For each dead operon pair, we show the gene order and the predicted or known operon structure in E. coli K12 and its relatives. The amount of spacing between genes is not shown. The trees show the branching order of the species according to the tree in Figure 2. We also show a parsimonious reconstruction of events, marked by “+” and “−” on the branches and the labels at right. Genes that are essential for growth in rich media (from [79]) are marked with an asterisk (*).

When we investigated *lysA–dapF* and *ribD–ribE* in detail, we discovered another mechanism of operon death, which we term “replacement.” *dapF* and *lysA* encode the final two steps of lysine synthesis, but *dapF*'s product is also essential for cell wall synthesis. In E. coli and some other species, *lysA* expression responds to lysine levels via an activator that is encoded by the adjacent gene *lysR* [37,38]. In many of its relatives, *lysA* is in an operon with *dapF* (see Figure 6, specifically Figure 6A) and is not regulated by lysine [38]. In phylogenetic analyses, *lysR*-associated *lysA* from diverse species constitutes a distinct clade (unpublished data), which we term *lysA2*. This suggests horizontal transfer, as does the presence of both *dapF–lysA* and *lysR–lysA2* in some species. Thus, the parsimonious reconstruction is that E. coli acquired *lysR–lysA2* by HGT and then deleted *lysA* (Figure 6A). Consistent with deletion of *lysA,* the predicted E. coli operon retains genes on both sides of the missing *lysA*. The putative operon is consistent with the reported transcription start well upstream of *dapF* [39] and with polar effects of upstream insertions on *xerC,* which is well downstream of *dapF* [40].

Similarly, *ribD* and *ribE* encode enzymes for the synthesis of riboflavin. It has been noted that many genomes have a second copy of *ribE* that lies outside of the ancestral operon [41], which we term *ribE2* (see Figure 6B). These *ribE2* genes form a distinct clade (unpublished data), and E. coli has only *ribE2*. Again, the parsimonious reconstruction is that *ribD–ribE* died when *ribE* was replaced by the horizontally acquired *ribE2*.

Given the distinction between *lysA* and *lysA2,* or between *ribE* and *ribE2,* are these genuine dead operons or are they errors in our automated analysis? We feel that the choice is somewhat arbitrary. Because *lysA*/*lysA2* and *ribE/ribE2* are believed to have the same function, we prefer to consider *lysA–dapF* and *ribD–ribE* as dead operons. We also note that these HGT events required detailed phylogenetic analysis to uncover, and hence that previous analyses of operon destruction, which examined events across much larger phylogenetic distances (e.g., [7]), probably included similar cases.

Is operon death by replacement a common mechanism? To study this question systematically, we asked whether genes in dead operons were more likely than genes in live operons to have paralogs or to show evidence of HGT. We identified paralogs across 61 completely sequenced *γ*-Proteobacteria by using the COG database [36]. Although we required all genes in both the dead and the live operons to lack paralogs in *E. coli,* we can still ask if paralogs are common in other organisms. On average, genes in dead operons had paralogs in 10.2% of the genomes, which is statistically indistinguishable from the rate of 9.4% for genes in live operons (*p* > 0.5, *t* test). We also built phylogenetic trees for all 118 genes in dead operons and compared the resulting trees with the species tree of [42] (see Materials and Methods). We found no evidence of HGT for most of these genes (*p* > 0.05 for 90.0% of genes, Kishino–Hasegawa test). Thus, we suspect that operon death generally occurs by genome rearrangements, or perhaps by insertions that are masked by later rearrangements, and not by replacement.

#### Rapid death of new operons and of operon pairs with unrelated functions.

Why do operons die? As a first step toward answering this question, we compared the death rates of different types of operons. For example, do operons that contain genes in different COG functional categories have different likelihoods of dying? For each of the 14 functional categories with at least ten genes in the combined dataset of live and dead operons, we performed a Fisher exact test, and to correct for multiple testing we used the false discovery rate with a cutoff of 0.05. We found unusually high survival rates for energy production and conversion operons (31 genes in surviving operons versus zero in dead operons). We found unusually low survival rates for coenzyme metabolism operons (20 genes in surviving operons versus 19 in dead operons) and for amino acid transport/metabolism operons (24 genes in surviving operons versus 16 in dead operons). We speculate that the regulation of amino acid and coenzyme metabolism might evolve quickly to reflect the nutrients present in a particular niche.

**Figure 7:**
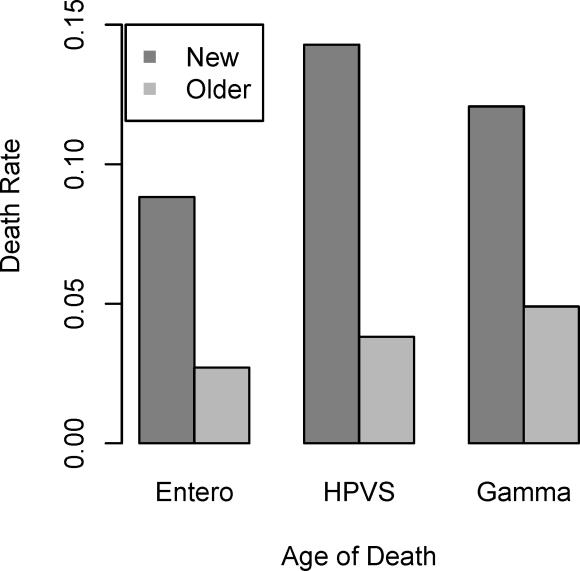
New Operons Die at Faster Rates Ancestral operons were identified by their presence in two or more consecutive groups of relatives, and were considered dead if they were no longer in the same operon in E. coli K12. The death rate at a given “age” is the proportion of operons that are present in that group but not in more recent relatives. Here, an operon is considered new at the time of its death if it is present only in the minimum two consecutive groups. In increasing order, the ages are “Entero”—Enterobacteria other than E. coli or *Salmonella;* “HPVS”—*Haemophilus, Pasteurella, Shewanella,* and *Vibrio* species; and “Gamma”—other *γ*-Proteobacteria. All differences between new and older operons were statistically significant (*p* < 0.05, Fisher exact test).

We also found that new operons are much more likely to die than are older operons (Figure 7). However, even among ancient operons that are conserved between the *β*- and *γ*-Proteobacteria, 14% are shuffled apart in E. coli K12. Not surprisingly, operon pairs with conflicting COG function codes [36] are more likely to die (25% versus 10%, *p* < 0.005, Fisher exact test). Even ancient operons are more likely to die if they have distinct COG function codes (32% versus 8%, *p* < 0.001, Fisher exact test). These results raise the question of why these functionally incoherent operons arose in the first place.

### Operon Evolution Alters Gene Expression

**Figure 8:**
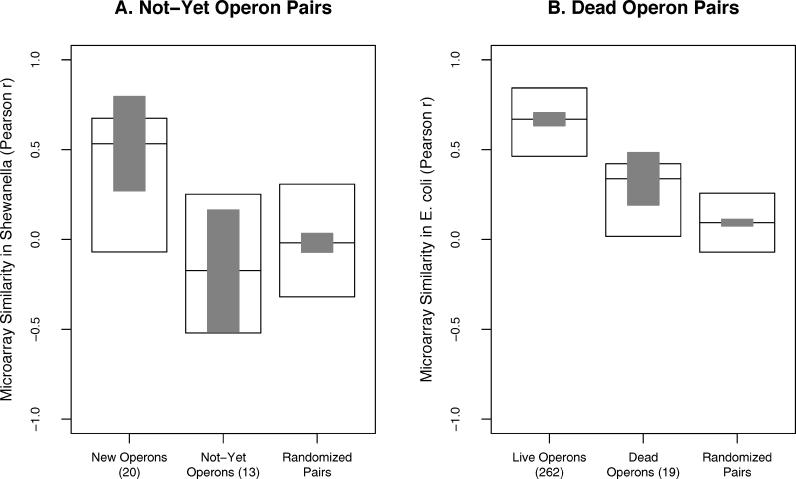
Operon Evolution Affects the Pattern of Gene Expression (A) The distribution of microarray similarity in Shewanella oneidensis MR-1 for new E. coli operon pairs that had “already” formed in *Shewanella,* for “not-yet” pairs that are far apart in *Shewanella* but are in newer operons in *E. coli,* and for randomized pairs of the genes in the latter pairs. For each distribution, the box shows the median and first and third quartiles, and the grey bar shows a 90% confidence interval for the median, so that if two bars do not overlap then the difference in medians is significant (*p* < 0.05). (B) The distribution of microarray similarity in E. coli K12 for “live” new operon pairs that are conserved in *Shewanella,* for “dead” operon pairs of similar age that are far apart in E. coli K12, and for randomized pairs of the latter genes. For both (A) and (B), *t* tests gave similar results for significance (unpublished data).

If operon formation is driven by gene expression, then operon formation should be associated with changes in the expression patterns of the constituent genes. Although we cannot study gene expression patterns in the ancestors of *E. coli,* we can study the expression patterns of orthologous genes in a related bacterium that diverged before the operon formed. We examined operon pairs that formed in the E. coli lineage soon after its divergence from Shewanella oneidensis MR-1, which we refer to as “not-yet” operons in *Shewanella*. We compared the co-expression of these pairs to that of pairs that formed new operons just *before* the divergence (pairs that are “already” in operons in *Shewanella*). In *Shewanella,* the “not-yet” operon pairs are not co-expressed, while the “already” operon pairs are, not surprisingly, co-expressed (Figure 8A). Hence, operon formation has a major effect on gene expression patterns.

To see if operon destruction also leads to changes in gene expression, we compared the co-expression of conserved operon pairs with that of “dead” operons of the same evolutionary age that are split apart in E. coli K12 (see Materials and Methods). We found that dead operons were significantly less co-expressed than operons that were still alive, but significantly more co-expressed than random pairs (Figure 8B). We also examined the expression of dead operon pairs in B. subtilis, and again found that they were much less co-expressed than the live operon pairs (Figure S4). Thus, operon destruction also has a major effect on gene expression patterns, but it does not entirely eliminate the similarity of expression.

### Operon Destruction Can Be Associated with Adaptive Changes in Gene Expression

If operon evolution leads to large changes in gene expression patterns, are these changes adaptive? Because operon formation often brings functionally related genes together, it seems unlikely to be a neutral process. On the other hand, operon destruction has been described as a neutral process [7]. In particular, one might expect that the destruction of functionally related operons would be deleterious, because as shown in the previous section, it causes the genes to have different expression patterns.

When we examined dead operon pairs, however, we found some cases of adaptive changes in gene expression. First, *ruvC–ruvA–ruvB* encode a DNA repair complex [43]. We reconstructed the evolutionary history of this operon and its regulation by using previous computational and experimental studies [45–47] and by comparing this regulation to the species tree. As shown in Figure 6C, these genes form an ancestral operon that is regulated by LexA, which is the major regulator of DNA repair, in distant relatives of E. coli. In *E. coli,* however, only *ruvA–ruvB* are regulated by LexA and *ruvC* is transcribed separately. The most parsimonious explanation for these differences in regulation and operon structure is that the LexA regulation of *ruvCAB* was lost in the E. coli lineage, so that none of these genes were regulated by LexA. Then, *ruvA–ruvB* acquired a LexA-regulated promoter (e.g., in *Vibrio*), so that *ruvA–ruvB* but not *ruvC* were regulated by LexA. In *E. coli, ruvAB* was further separated from *ruvC* by the insertion of *yebC*. LexA regulation of *ruvA* and *ruvB* appears to be adaptive, as it makes biological sense and has evolved independently in ancestral (or distantly related) Proteobacteria and also in the *E.coli*/*Vibrio* lineage. Thus, the destruction of the *ruvCAB* operon is associated with an adaptive change in the regulation of *ruvAB*.

Second, as we discussed previously, *dapF–lysA* are in an ancestral operon, but in *E. coli, lysA* has been replaced by *lysA2,* which is regulated by LysR. Regulation of *lysA2* by LysR is believed to be an adaptive mechanism for lysine homeostasis. In the most parsimonious evolutionary scenario, *lysR–lysA2* was independently acquired by two lineages (Figure 6A), which also suggests that this arrangement is adaptive. As the ancestral operon contains genes that are essential for growth even when lysine is externally supplied *(dapF* and *xerC),* this regulation could not have evolved within the original operon. Our other example of operon death by replacement, *ribD–ribE,* also involves replacing a gene in an essential operon (but the regulation of *ribE2* is not known).

These examples illustrate that, surprisingly, the destruction of operons that encode genes with a close functional relationship can be accompanied by adaptive changes in gene expression. However, we do not know if the operon destruction itself was adaptive. More precisely, we do not know if operon destruction became fixed in a population together with the adaptive change in gene expression, or if the operon destruction became fixed first by a neutral process.

## Discussion

### Adaptive Evolution of Operons

Several of our findings suggest that the evolution of operons may be adaptive. First, both the birth and the death of operons lead to large changes in expression patterns. Gene expression is believed to be under strong selection in *E. coli:* the majority of known regulatory sequences are highly conserved [35], genes are often regulated by multiple transcription factors [48], gene expression patterns show convergent evolution in the wild [49] and in laboratory experiments [50], and gene expression levels can evolve to optimality in laboratory experiments [33]. Thus, we argue that these changes in operon structure are also under strong selection. Second, some operons acquire several new genes in a relatively short period of evolutionary time; this accelerated evolution suggests positive Darwinian selection. Third, highly expressed operons are particularly likely to contain wide internal spacings and internal regulatory elements, which can be explained by strong selection to avoid making large amounts of unnecessary protein. Finally, many new operons contain ORFan genes downstream of native genes, which may reflect selection for expression of the ORFan genes.

These results contrast to a previous suggestion that selection to maintain operon structure is weak, so that genome rearrangements cause neutral or slightly deleterious turnover of operon structure [7]. The two explanations of neutral and adaptive evolution are not exclusive—the formation and death of operons could be nearly neutral in some cases and highly adaptive in others. Intensive analysis of specific operons will be required to distinguish these possibilities. We have discussed two examples, the replacement of *lysA* in the *dapF* operon with a LysR-regulated *lysA* and the regulation of *ruvA–ruvB* by LexA, in which the accompanying change in regulation appears to be adaptive. However, even here the change in operon structure could have been fixed neutrally before the regulatory change occurred.

Both *lysA* and *ruvA–ruvB* were probably in ancestral operons that contained essential genes (Figure 6). Similarly, in the second case of operon death by replacement that we identified, *ribD–ribE,* it again appears that the ancestral operon contained an essential gene (*nusB;* see Figure 6B) and hence must have been constitutive or growth-regulated. Thus, the turnover of operon structure may accompany switching between constitutive and inducible expression. Although constitutive expression may seem deleterious, it could be neutral if the capability is often required, and could be adaptive if lack of the protein would create delays in growth until large amounts of new protein were synthesized. Such “just-in-case” or “standby” expression of proteins that are not required for rapid growth appears to be common in the soil bacterium B. subtilis [51].

### Non-Optimal Evolution of Operons

If operon evolution is adaptive, then do operons reach an optimal arrangement? In general, we do not know what would make a gene regulatory system optimal, and we often do not know what the criteria are. However, for inducible biosynthetic capabilities such as amino acid synthesis, a plausible design goal is to produce product quickly, so that growth can resume, while also minimizing the amounts of enzyme synthesized. For simple (linear) metabolic pathways, optimal design by this criterion requires differential timing of gene expression, with earlier induction for genes that encode the first steps in the pathway [52]. This suggests that placing genes in operons may prevent fine-tuning of the timing of induction, and could be inherently suboptimal. Operons might exist despite this disadvantage because they facilitate the evolution of co-regulation [14].

Constraints on how operons evolve are also likely to lead to non-optimal operons. We have already discussed how the introduction of a LexA-regulated promoter between *ruvC* and *ruvA–ruvB* was adaptive, but the regulation of all three genes by LexA would, we imagine, be more adaptive. More broadly, operons containing native genes often form by deletion, so that the two genes in the new operon need to have been near each other and on the same strand. Although there is some tendency for genes with similar functions or expression patterns to cluster together on a larger scale than operons [17,53], it seems unlikely that the optimal partners for new operons will be found near each other. Thus, we would not expect new operons that formed by deletion to be optimal.

Similarly, the formation of new native–ORFan operon pairs may be driven by selection for the presence of the ORFan rather than for optimal regulation. This is because selection for the presence or absence of a gene should be much stronger than selection on its regulation. As the insertion of any particular ORFan is probably very rare, the operon that forms and becomes fixed in the population might not be the optimal one. Furthermore, optimal regulation of the ORFan may not be available from any of the pre-existing native promoters.

### Generality of Our Findings

Although our analysis focused on *E. coli,* we observed similar patterns of operon evolution in B. subtilis. More broadly, across almost all bacteria and even archaea, operons are characterized by close spacing of genes within operons, modest conservation, and modest functional similarity [1–3,5,8]. As our findings elaborate on these patterns and why they exist, our findings seem likely to generalize to most prokaryotes. However, some organisms clearly have different patterns of operon evolution, such as genome shuffling and wide spacing within operons in *Synechocystis* [3,5,7,8]. Another difference is that in some archaea, the first genes of operons often have no 5′ untranslated region, so that the translation start is the transcription start [54,55]. Finally, we note that E. coli and its relatives have undergone slower evolution of gene order than other groups of bacteria [11], which likely facilitated our analyses. As more genome sequences and genome-wide datasets become available, it will be easier to examine operon evolution in other groups of prokaryotes.

### Consequences for Genome Annotation

On a practical note, the lack of co-expression of “not-yet” operons extends previous observations that many new operons contain genes with unrelated functions [4,14]. Our observation that newer operons have high death rates also confirms that the genes in them may not be functionally related. Thus, we caution that the presence of a gene in an operon is not a strong indicator of its function unless the operon is well-conserved. Of the new operon pairs that are new to the Enterobacteria and contain two annotated genes, only four out of nine have related functions according to COG [36]. This statistic probably overstates the chance of two genes in a new operon being related, as there are many uncharacterized genes, and it is more likely that both genes in an operon will be characterized if they have closely related functions.

As examples of how over-reliance on new operons can lead to incorrect annotation, consider *flhE* and *btuE*. The new operon *flhBAE* combines native genes *flhA* and *flhB,* which are required for flagellar export, with the ORFan *flhE,* which is not [56,57]. Nevertheless, *flhE* is often annotated as a flagellar gene, apparently purely because of its location. Another new operon unique to the Enterobacteria, *btuCED,* includes two components of the vitamin B12 ABC transporter and also *btuE,* which is not required for vitamin B12 transport [58]. Instead, *btuE* belongs to the glutathione peroxidase family and is not homologous to ABC transporters (unpublished data). Nevertheless, *btuE* is often mis-annotated as a vitamin B12 ABC transporter in sequence databases.

Most automated predictions of gene function are not affected by these issues because they use only highly conserved operons [6,59], but operon predictions based on the distance between adjacent genes have been used to aid in function prediction [60]. The latter method was validated by testing it against textual gene annotations, but the over-annotation of new operons, as with *flhE* and *btuE,* could possibly have exaggerated its benefit. In any case, we suspect that automated function predictions could be improved by down-weighting evidence from the newest operons.

## Materials and Methods

### Operons.

For more than 100 genomes, we predicted whether pairs of adjacent genes that are on the same strand are co-transcribed based on the intergenic distance between them, whether orthologs of the genes are near each other in other genomes, and the genes' predicted functions [3]. Both the predictions and the underlying features are available at http://www.microbesonline.org/operons [61]. These operon predictions are more than 80% accurate on pairs of genes in diverse prokaryotes, based on databases of known operons and on analysis of microarray data. For analyses of operon spacing, we used a database of known E. coli K12 operons [48] instead of predictions.

### Evolutionary history of E. coli genes and operon pairs.

For genes and operons in *E. coli,* we used the evolutionary analysis of [14]. Briefly, we divided the sequenced prokaryotes into groups at varying evolutionary distances from E. coli K12—1) other strains of E. coli and *Shigella*, 2) *Salmonella* species, 3) other Enterobacteria, 4) allied *γ*-Proteobacteria (*Haemophilus, Pasteurella, Vibrio,* and *Shewanella* species), 5) distant *γ* -Proteobacteria (*Pseudomonas, Xanthomonas,* and *Xylella* species), 6) *β*-Proteobacteria, 7) other Proteobacteria, and 8) non-Proteobacteria, including Archaea. E. coli K12 genes that had at least one homolog from BLASTp or from COG [36] in each of the groups 1–7 were considered native. (Genes were assigned to COGs by reverse position–specific BLAST [62] against CDD [63].) Genes that had no homologs in two consecutive groups, but had homologs in a more distant group, were considered HGT. Genes that had no homologs any of groups 6–8 were considered ORFans, and the most distant group that did contain a homolog of each ORFan was used an estimate of the ORFan's evolutionary age. (Most ORFans were present in all groups leading up to the oldest group.) Genes that did not meet the criteria to be native, HGT, or ORFan were considered unclassifiable; prophages and transposons were also excluded.

We classified operon pairs in a similar way. For each pair of adjacent E. coli K12 genes that were predicted to be in the same operon, we asked which groups of genomes contained homologous operons. To account for the frequent reordering of genes in operons [7], we did not require the homologs to be adjacent, but only that they be in the same predicted operon. Operons of age four or less were considered new, operons absent in two consecutive groups and present in a more distant group were considered HGT, and operons present in each of groups 1–7 were considered old. In previous work, we validated the process of assigning ages to genes and operons by showing that HGT operons generally contained HGT genes of the same age as the operon [14].

Because our operon prediction method takes conservation of gene order into account, it may be less likely to predict some new operons. However, lack of conservation is weak evidence against operon-ness, and the method does identify many new operons. Conversely, because many other genomes are considered, false negative operon predictions in other genomes should not lead to the spurious identification of “new” operons that are not actually new. (See [14] for more discussion of this point and other validation of the new operon pairs).

To identify dead operons in *E. coli,* we first enumerated all pairs of E. coli K12 genes that were orthologous to predicted operon pairs from any other genome. Here for orthologs we used either bidirectional BLASTp hits with 75% coverage or genes in the same COG. We retained pairs that were predicted to be in an operon in two consecutive groups (e.g., both a group 4 genome and a group 5 genome). The requirement for the operon to be present in two consecutive groups should eliminate false positives from the operon predictions; indeed, most non-operon pairs will no longer be near each other on this evolutionary timescale. Of these pairs, those that were adjacent in E. coli K12 and predicted to be in the same E. coli operon were considered “live” operons; pairs that were not in the same run of genes on the same strand (i.e., not in a candidate operon) were considered to be “dead” operons; and other pairs were considered ambiguous and discarded. Furthermore, we realized that if the ancient operon AB died, and gene B has a paralog B′, then both AB and AB′ can appear to be dead operons. To overcome this problem, we required that both genes be a unique member of their COG in E. coli K12, and furthermore that, in at least one member of the oldest outgroup, the bidirectional best hits of the E. coli genes be in the same operon. Manual inspection of the results found that this rule was effective. The functional relatedness (Table 3) and modest co-expression (Figure 8B) of many of the dead operon pairs also suggest that this analysis was accurate.

For the co-expression analysis of “not-yet” operons in S. oneidensis (Figure 8A), we began with adjacent genes that are predicted to be in the same operon in *E. coli,* that were not classified as HGT operons, and that had similar expression patterns in E. coli (Pearson *r* ≥ 0.5). We further required the genes to have orthologs (bidirectional best hits with 75% coverage and 40% amino acid identity) in S. oneidensis. For the “not-yet” pairs we required that there be at least four intervening genes, while for the “already” operon pairs we required the genes to be adjacent.

### Evolutionary history of B. subtilis genes and operon pairs.

We performed a similar evolutionary analysis for B. subtilis. The groups of more distantly related bacteria were 1) *B. licheniformis,* the Bacillus cereus group, and Geobacillus kaustophilus HTA426; 2) *Bacillus clausii, Bacillus halodurans,* and Oceanobacillus iheyensis; 3) *Listeria* species; 4) *Staphylococcus* species; 5) lactic acid bacteria, including *Streptococcus, Lactobacillus,* and *Enterococcus* species; 6) Other Firmicutes, including Mollicutes (e.g., *Mycoplasma*), Clostridia, and *Symbiobacterium thermophilum;* and 7) other bacteria and archaea. This choice of outgroups was strongly supported by whole-genome trees (unpublished data) and is consistent with accepted phylogenies, except perhaps for *Symbiobacterium,* which we found, in accord with [64], to be in the Firmicutes and not in the Actinobacteria. Genes and operons of age four or less were considered new.

For a dataset of known operons in *B. subtilis,* we combined the operons collated from the literature by [7] and [24].

### Microarray data.

To quantify the similarity of two genes' expression patterns, we used the Pearson correlation of their normalized log ratios across microarray experiments. For *E. coli,* we combined data from the Stanford Microarray Database (SMD) [65], from ASAP [66], and from Covert et al*.* [67]. For *B. subtilis,* we used data from SMD. For both organisms, we used only experiments that measured or compared mRNA levels. For the SMD data, we began with the normalized log-ratios provided by SMD, and then subtracted the mean from each experiment. For the other datasets, we subtracted the mean from each gene's log-level, to give “normalized” log-levels for each experiment, and then subtracted the mean for each gene across experiments, to give normalized log-ratios. For the Covert et al. data, which included values of zero, we added a small amount (five) before taking logarithms. For S. oneidensis MR-1, we used data on salt stress [68], heat shock [69], high and low pH stress [70], strontium stress [71], and cold shock (Z. He, Q. He, and J. Zhou, unpublished data); these were normalized as previously described [70]. The data for all three species is available as Dataset S1.

To quantify gene expression (mRNA) levels in E. coli K12, we used the average foreground intensity across arrays and across both red and green channels in the SMD data. We used intensities rather than more direct measures of expression levels, which can be obtained from microarray experiments where an mRNA sample is compared with genomic DNA, because only a few of the experiments were of that type. Within the “genomic control” experiments, the average across replicates of the intensity in the mRNA channel was highly correlated with the average log-ratio between the mRNA and genomic DNA channels (the Spearman rank correlation was 0.84). We also note that this measure of expression level was highly correlated for operon pairs (Spearman rank correlation = 0.77), which is significantly higher than the rank correlation for CAI (0.55). mRNA levels in B. subtilis were quantified by the same method.

### Testing for accelerated evolution.

As shown in Figure 4, new operon pairs are often adjacent to other new operon pairs of the same age. To see how often this would occur under completely random evolution, we used the fraction *p_i_* of operon pairs that have age *i,* the fraction *q* of operon pairs that are adjacent to the next (downstream) operon pair, and the total number *N* of operon pairs. (Operon pairs AB and BC within the operon ABC are adjacent, while the standalone operon AB is not adjacent to another operon pair.) Under random evolution, each operon's age and adjacency status would be chosen randomly and independently of any adjacent pair's age, so that the number of adjacent new operon pairs of the same age would be

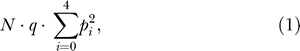
and the number of adjacent new operon pairs would be

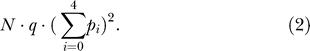



### Modifications to pre-existing operons.

To identify and classify the new operon pairs that arose by modification to pre-existing operons, we performed an automated analysis (shown in Figure 4B) and also inspected the results manually (Table S1). The automated analysis relied on comparing the ages of the new operon pair with the age of adjacent or surrounding operon pairs. For example, if the operon pair AB was prepended to the pre-existing operon BC, then AB should be newer than BC. If the operon ABC arose from inserting the gene B into the pre-existing operon AC (or from replacing gene D in the operon ADC), then both AB and BC should be newer than AC. If the operon ABCD formed by joining two pre-existing operons AB and CD, then BC should be newer than either AB or CD. To avoid confusion due to paralogs, we only considered pairs where the age using homologs from COG matched the age using putative orthologs (bidirectional BLASTp hits). Manual inspection was performed with the MicrobesOnline comparative genomics browser at http://microbesonline.org [61], with careful attention to cases where potential orthologs were not identified automatically.

### Phylogenetic trees.

The tree of E. coli and its relatives (Figure 2) was computed from the concatenated protein sequences of 15 ubiquitous single-copy COGs. These were aligned with MUSCLE [72]; positions with gaps or adjacent to gaps were removed; and a tree was constructed with TreePuzzle 5.1 [73], using gamma-distributed rates. The rooting reflects accepted phylogenies.

To test genes in dead E. coli operons for evidence of HGT, we compared the phylogenetic tree inferred from the protein sequences with the species tree of [42]. From orthologs (bidirectional best BLASTp hits) among the species in the species tree, we constructed protein sequence alignments with ClustalW [74] and the BLOSUM80 matrix, we removed columns containing gaps, and we constructed phylogenetic trees with TreePuzzle 5.1 [73], using the default settings. To see if the resulting tree was consistent with the species tree, we used the one-sided Kishino–Hasegawa test recommended by [75]. High *p*-values indicate accepting the species tree.

### Statistics.

Statistical tests were conducted with the R Project for Statistical Computing open-source statistics package (http://www.r-project.org).

## Supporting Information

Dataset S1 Microarray Data for Three Species (7.0 MB ZIP)

Figure S1 Types of New B. subtilis Operons (A) Types of genes in new operon pairs and in other operon pairs.
(B) Types of new operon pairs. Only new operon pairs involving native and ORFan genes are shown (there are relatively few HGT genes in the new operons).
(C) Microarray co-expression of predicted new operon pairs of each of the three major types. As a negative control, we also tested non-operon pairs (adjacent genes on the same strand that are believed not to be co-transcribed).
(8 KB EPS)

Figure S2 Spacings between Adjacent Genes in the Same Operon of B. subtilis
(A) Known operon pairs in B. subtilis often have different spacing than the orthologous operon in B. licheniformis. For each class of spacing in E. coli (*x-*axis), a vertical bar shows the proportion with various amounts of change.
(B) The frequency of different types of spacings for operon pairs classified by their evolutionary history (left), their expression level as estimated from microarray data (middle), or whether the operon has an alternative transcript (right). Because operon predictions rely heavily on spacing, only known B. subtilis operons were used.
(C) The distribution of microarray similarity for known operon pairs spaced by less than 50 bp or by more than 50 bp and for alternatively transcribed operon pairs. Operons that are known to be alternatively transcribed were excluded from the “narrow” and “wide” sets.
(12 KB EPS)

Figure S3 Accelerated Evolution of Some B. subtilis Operons New operon pairs are more likely to be adjacent to each other than expected by chance. The surplus of adjacent pairs of the same age is particularly striking. The error bars show 95% confidence intervals from a χ^2^ test of proportions.
(4 KB EPS)

Figure S4 Dead Operon Pairs in B. subtilis Are Moderately Co-Expressed For each distribution, the box shows the median and first and third quartiles, and the grey bar shows a 90% confidence interval for the median, so that if two bars do not overlap then the difference in medians is significant (*p* < 0.05).
(4 KB EPS)

Protocol S1 New Operons that Formed by Deletion (22 KB PDF)

Table S1 Modifications to Pre-Existing Operons (31 KB DOC)

## Abbreviations

COGs: conserved orthologous groups
HGT: horizontal gene transfer
ORFan: open reading frame that lacks homologs outside of a closely related group of organisms
SMD: Stanford Microarray Database
