# Correction: The Life-Cycle of Operons

**DOI:** 10.1371/journal.pgen.0020126

**Published:** 2006-07-28

**Authors:** Morgan N Price, Adam P Arkin, Eric J Alm

DOI: 10.1371/journal.pgen.0020096

In *PLoS Genetics*, volume 2, issue 6:

The second of the authors' institutional affiliations was incorrectly listed as Virtual Institute for Microbial Stress and Survival, University of California San Francisco, San Francisco, California, United States of America.

The correct affiliation is Virtual Institute for Microbial Stress and Survival, Lawrence Berkeley National Laboratory, Berkeley, California, United States of America.

