# Supplementary material for: The Life-Cycle of Operons
Source: PLoS Genet. 2006 Jun 23;2(6):e96. doi: 10.1371/journal.pgen.0020096 (PMC1480536; doi:10.1371/journal.pgen.0020096)
Supplement: Supplementary file 1 [file pgen.0020096.sd002.pdf]

Protocol S1: New Operons that Formed by Deletion

If two genes that are in the same operon in *E. coli* are near each other but in different operons in *Vibrio* species, then we infer that the operon formed by deleting the intervening genes. A second possible explanation is that the common ancestor of the Enterobacteria and *Vibrio* formed the operon, and that another gene was then inserted in the *Vibrio* lineage. Finally, a third alternative is that the common ancestor formed nearby genes by rearrangement, without forming an operon, and then both insertions in the *Vibrios* and deletions in the *E. coli* lineage occurred. The deletion scenario is more parsimonious than the insertion scenario because it involves a single operon creation/destruction event, instead of operon creation followed by later destruction in the *Vibrios*. The deletion scenario is more parsimonious than the insertion/deletion scenario because fewer events are required.

Conserved proximity in *Shewanella oneidensis* MR-1 occurs for two of the putative deletion events. First, *serB* and *radA* (also known as *sms*) are separated in *S. oneidensis* by a homolog of *VC2344* and one additional protein. Second, *ygiF* and *glnE* are separated by only 8 intervening genes in Shewanella, including an ortholog of *VP0422*. Because *S. oneidensis* probably diverged from *E. coli* before the *Vibrios* (the quartet puzzling score was 97/100), this shows that the common ancestor of the *Vibrios* and *E. coli* had the intervening genes and not the operon, as in the deletion scenario.

If the deletion scenario is correct then the intervening genes should be absent from the Enterobacteria and sometimes present in more distant relatives of *E. coli*. In the two cases of conserved proximity in *S. oneidensis*, an intervening gene is present in the same location in *S. oneidensis* and absent from Enterobacteria and from other closer relatives of E. coli such as *Haemophilus* and *Pasteurella*. Most of the other intervening genes appear to be horizontally transferred into *Vibrio* from distant bacteria, so that their absence from the Enterobacteria is unsurprising and uninformative. A striking exception is *asnC*, one of the genes that separates *prlC* and *yihQ* in Vibrios: *asnC* has clear orthologs in most Enterobacteria and in *S. oneidensis*. Although this type of deletional rearrangement seems somewhat surprising, it is equivalent to insertional rearrangements (as in the formation of *ptr-recB*, see Table 1) and is arguably more parsimonious than the alternative, which would be rearrangement to form the operon and then an insertion.

Another argument for deletion arises with *btuB* and *murI*: the 68 bp overlap results from the addition of over 20 amino N-terminal amino acids to *murI* that is not present in genes without the operon. These amino acids are encoded by the 3' end of *btuB*. This overlap is probably correct because the predicted molecular weight from the *murI* sequence matches that observed in Western blots (P. Doublet et al., J. Bacteriol. 175:2970-9). Without the overlap the gene product would be 10% too light. The overlap is present in all of the sequenced Enterobacteria, and is probably the ancestral state of the operon. Thus, we speculate that the original start codon was lost during the deletion event. The original start codon could also have been lost by a rearrangement to create the operon, followed by an insertion in *Vibrio*, but then it would be particularly difficult to insert the *Vibrio* ATPase between *btuB* and *murI*.
