# Supplementary material for: The Life-Cycle of Operons
Source: PLoS Genet. 2006 Jun 23;2(6):e96. doi: 10.1371/journal.pgen.0020096 (PMC1480536; doi:10.1371/journal.pgen.0020096)
Supplement: Supplementary file 3 [file pgen.0020096.st001.doc]

Table S1.

Modifications to pre-existing operons.

**Join Two Pre-existing Operons**

*yggS-yggT-yggU-rdgB-yggW*

*mhpA-mhpB-mhpC-mhpD-mhpF-mhpE*

***yiaKL****-yiaMNO-****lyxK****-sgbH-sgbU-sgbE*

**Internal Replacement**

*ymcC-****ymcB****-ymcA*

*rpoZ-spoT-****spoU****-recG*

*nirB-nirD-****nirC****-cysG*

**Internal Insertion**

*celA-celB-celC-****celD****-celF-ydjC*

**Prepend (5 known / 12 total**)

*mhpA-mhpB-****mhpC****-mhpD-mhpF-mhpE*

***csgD****-csgE-csgF-csgG*

*damX-****dam****-rpe-gph-trpS*

*nfrA-****nfrB****-nfrC-nfrD-nfrE-nfrF-nfrG*

***creA****-creB-creC-creD*

**Append (5 known / 15 total**)

*cynT-cynS-****cynX***

*pspA-pspB-pspC-****pspD****-pspE*

*fucP-fucI-fucK-fucU-****fucR***

*yhdT-panF-****prmA***

*mtlA-mtlD-****mtlR***

The modified operons analyzed in Figure 4B were examined by hand. To avoid potential false positives in the operon predictions, only coexpressed pairs (Pearson correlation > 0.5) were included, and known non-operon pairs were excluded. Because of the large number of append and prepend events, only known operons (, ) affected by those events were examined. Pre-existing operons are underlined; inserted, replaced, appended, and prepended genes are in bold.
